# Characterization of Oogonial Stem Cells in Adult Mouse Ovaries with Age and Comparison to In Silico Data on Human Ovarian Aging

**DOI:** 10.1089/scd.2022.0284

**Published:** 2023-03-03

**Authors:** Julie A. MacDonald, Hannah C. Sheehan, Andrew Piasecki, Luciana R. Faustino, Charlotte Hauschildt, Victor Stolzenbach, Dori C. Woods, Jonathan L. Tilly

**Affiliations:** Laboratory of Aging and Infertility Research, Department of Biology, Northeastern University, Boston, Massachusetts, USA.

**Keywords:** germ line stem cell, oogonial stem cell, SSEA1, Dppa3, meiosis, oocyte, ovary, aging

## Abstract

Many adult somatic stem cell lineages are comprised of subpopulations that differ in gene expression, mitotic activity, and differentiation status. In this study, we explored if cellular heterogeneity also exists within oogonial stem cells (OSCs), and how chronological aging impacts OSCs. In OSCs isolated from mouse ovaries by flow cytometry and established in culture, we identified subpopulations of OSCs that could be separated based on differential expression of stage-specific embryonic antigen 1 (SSEA1) and cluster of differentiation 61 (CD61). Levels of aldehyde dehydrogenase (ALDH) activity were inversely related to OSC differentiation, whereas commitment of OSCs to differentiation through transcriptional activation of *stimulated by retinoic acid gene 8* was marked by a decline in ALDH activity and in SSEA1 expression. Analysis of OSCs freshly isolated from ovaries of mice between 3 and 20 months of age revealed that these subpopulations were present and persisted throughout adult life. However, expression of *developmental pluripotency associated 3* (*Dppa3*), an epigenetic modifier that promotes OSC differentiation into oocytes, was lost as the mice transitioned from a time of reproductive compromise (10 months) to reproductive failure (15 months). Further analysis showed that OSCs from aged females could be established in culture, and that once established the cultured cells reactivated *Dppa3* expression and the capacity for oogenesis. Analysis of single-nucleus RNA sequence data sets generated from ovaries of women in their 20s versus those in their late 40s to early 50s showed that the frequency of *DPPA3*-expressing cells decreased with advancing age, and this was paralleled by reduced expression of several key meiotic differentiation genes. These data support the existence of OSC subpopulations that differ in gene expression profiles and differentiation status. In addition, an age-related decrease in *Dppa3*/*DPPA3* expression, which is conserved between mice and humans, may play a role in loss of the ability of OSCs to maintain oogenesis with age.

## Introduction

Since the initial report of the existence of mammalian female germ line or oogonial stem cells (OSCs) and postnatal oogenesis in mouse ovaries in 2004 [1], tremendous progress has been made in elucidating the properties and functions of these tissue-specific adult stem cells in female reproductive biology across a variety of mammalian species (reviewed in Martin et al. [2]), including humans [3–15]. Among many key observations made over the years, intraovarian transplantation-based approaches with mice and rats have been used to establish the capacity of OSCs to generate functional oocytes that fertilize to produce embryos and viable offspring in vivo [3,16–22].

Recently, the generation of fully functional oocytes from mouse OSCs incorporated into ovarian organoids reconstituted in vitro has also been demonstrated [23]. In parallel, rigorous genetic studies with mice using both suicide gene technology and lineage tracing have firmly established the importance of ongoing postnatal oogenesis to adult ovarian function and natural female fertility under physiological conditions [21,24].

In studies of adult human ovarian tissues, it has been reported that OSCs persist in postmenopausal women and that the cells can reactivate meiotic differentiation into oocytes when reintroduced into human ovarian cortical tissue in vitro or when cultured under defined conditions ex vivo [5,7,13]. These observations are consistent with prior studies with mice that showed that de-novo oogenesis resumes in aged ovarian tissue after its reintroduction into a young adult ovarian environment in vivo [25]. While these data collectively support that OSCs enter a reversible quiescent state as females age, little else is known about the changes that occur in these specialized stem cells during the course of adult life in any mammalian species.

Recently, it has been proposed that OSCs undergo a gradual shift toward increased population heterogeneity as females age, and that this heterogeneity arises due to random genetic mutations that occur as a consequence of repeated cell divisions, accumulation of oxidative damage, or both, in individual OSC clones over time [26]. It is important to note, however, that almost all published studies of OSCs to date have viewed the cells as a relatively homogenous population of adult germ line stem cells, despite the fact that cellular heterogeneity has been documented in many adult somatic stem cells [27], including those that support hematopoiesis [28], neurogenesis [29], and cardiomyogenesis [30]. These somatic stem cell subpopulations exhibit stark differences in gene expression profiles, mitotic activity, and differentiation capacity, underscoring the need to define heterogeneity within adult stem cell subpopulations in the context of organ development, function, and failure.

Clarkson et al. [9] recently identified potential subpopulations of human OSCs based on differences in the levels of aldehyde dehydrogenase (ALDH) activity. Often used as a marker of stem and progenitor cells [31], ALDH activity is central to many processes that guide stem cell self-renewal and differentiation [32,33]. Other studies have identified expression of cluster of differentiation 61 (CD61; also referred to as integrin-β3 or ITGB3) in mouse and human OSCs [10], which is expressed in embryonic mouse primordial germ cells (PGCs) [34], but not in oocytes [35,36]. Although the precise function of CD61 in the female germ line remains unknown, its ectopic overexpression promotes the differentiation of germ cell-like cells from human umbilical cord mesenchymal stem cells [37]. It has also been reported that CD61 plays a key role in the specification of PGC-like cells (PGCLCs) from pluripotent stem cells in vitro [38].

To explore the possibility that OSCs are comprised of subpopulations that differ phenotypically and functionally, and that this heterogeneity may be important to consider in studies of how aging impacts OSC dynamics, here we characterized OSCs isolated from adult mouse ovaries by flow cytometry using antibodies to detect extracellular expression of the carboxy terminus of DEAD box helicase-4 (COOH-Ddx4) [3,4,39]. Although numerous studies have successfully sorted viable OSCs from the ovaries of diverse species using antibodies against COOH-Ddx4 [3,4,6–14,16–23,39] (see also supporting information table 1 in Alberico et al. [14] for a more complete listing of relevant publications), a few scientists have continued to claim that this OSC sorting protocol is flawed [40–42] (see also Woods and Tilly [43], Alberico et al. [14], and Woods and Tilly [44]).

Questions surrounding the isolation of OSCs with this approach were initially prompted by conclusions made many years ago that Ddx4 is a cytoplasmic protein in all germ cells [45,46], and thus, the protein should not be accessible to Ddx4 antibodies on the outer surface of OSCs to enable viable cell sorting (reviewed in Martin et al. [2]). However, these earlier studies on Ddx4 localization were published long before OSCs had been discovered. Using primary antibodies specific to either COOH-Ddx4 or the amino terminus of Ddx4 (NH_2_-Ddx4)—noting that COOH-Ddx4 is the region exposed on the extracellular surface of both OSCs [3,4,16,39] and PGCLCs specified from differentiating pluripotent stem cells [47,48] that enables viable cell sorting, prior studies of freshly isolated mouse OSCs demonstrated that COOH-Ddx4 is detectable in both fixed and permeabilized cells as well as in nonfixed and nonpermeabilized (viable) cells [3].

However, while fixed and permeabilized OSCs are also NH_2_-Ddx4 antibody-positive, nonfixed and nonpermeabilized OSCs are negative for Ddx4 when the amino terminus of the protein is targeted for analysis, confirming its intracellular localization. It should be emphasized that OSCs isolated using COOH-Ddx4 antibodies are NH_2_-Ddx4 antibody-positive as well if the isolated cells are fixed and permeabilized for the analysis. Thus, by using a different antibody directed to an entirely different region of the same protein, these findings establish that COOH-Ddx4-positive cells isolated from adult ovaries indeed express Ddx4 [3]. When viewed collectively, the results from this single-protein dual-epitope analysis of Ddx4 support that, in OSCs, the carboxy terminus of Ddx4 is externalized, whereas the amino terminus of the protein is internalized since it is only accessible to antibodies for detection if the cells are fixed and permeabilized before analysis.

In this study, OSCs isolated using COOH-Ddx4 antibodies were further analyzed for expression of CD61 as well as for expression of the primitive stem cell marker, stage-specific embryonic antigen 1 (SSEA1) [49]. Levels of ALDH activity were also assessed, and transcriptional activation of *stimulated by retinoic acid gene 8* (*Stra8*) was used as an established marker of primitive germ cell commitment to meiotic differentiation and oogenesis [50,51]. We further investigated if these endpoints were affected by estrogen [estradiol (E2)], which promotes the differentiation of OSCs into oocytes through transcriptional upregulation of *Stra8* [52], or by in vivo aging. Lastly, data obtained from the mouse studies were compared with results derived from analysis of single-nucleus RNA sequence (snRNA-seq) data sets derived from ovarian tissues of women in their 20s versus those in their late 40s to early 50s, which provide a comparative snapshot of global transcriptomic events associated with human female reproductive aging [53].

Our results show that OSCs, similar to other types of adult stem cells in the body, exist as subpopulations that differ in their cell-surface protein expression profiles and differentiation status. We also provide evidence of an evolutionarily conserved event that may underlie aging associated OSC quiescence, which revolves around changes in the expression of a key epigenetic modifier of germ cell differentiation.

## Materials and Methods

### Animals and reagents

Wild-type C57BL/6 female mice were obtained from Charles River Laboratories (Wilmington, MA) or from the Aged Rodent Colonies Program of the National Institute on Aging (Bethesda, MD; https://www.nia.nih.gov/research/dab/aged-rodent-colonies-handbook). All experimental procedures reported herein were reviewed and approved by the Institutional Animal Care and Use Committee of Northeastern University. Unless otherwise indicated, all reagents utilized were obtained from Thermo Fisher Scientific (Waltham, MA).

### Analysis of cultured OSCs

As previously detailed, OSCs were isolated from dissociated ovaries of young adult (2- to 3-month-old) female mice by fluorescence activated cell sorting (FACS) using a rabbit polyclonal antibody against COOH-Ddx4 and then established without feeder cells as actively dividing germ cell cultures [3,4,39]. To verify antibody specificity in targeting the carboxy terminus of Ddx4 during FACS, the following approaches were used. The initial sorting of OSCs from adult mouse ovaries was carried out using a rabbit polyclonal COOH-Ddx4 antibody from Abcam (Cambridge, MA; ab13840), as detailed previously [3,4,39]. Once the cells were established in culture and fully characterized as OSCs [3,4,39], the cells were reanalyzed by flow cytometry using two other Ddx4 antibodies: a custom-generated rabbit polyclonal COOH-Ddx4 antibody from Abgent (San Diego, CA) [54] and a rabbit polyclonal antibody against the NH_2_-Ddx4 (OAAB12716; Aviva Systems Biology, San Diego, CA).

For some experiments, the OSCs were comparatively analyzed as nonfixed and nonpermeabilized (viable) cells versus fixed and permeabilized cells to assess epitope localization (see the Immunolabeling of Viable Versus Fixed and Permeabilized OSCs section). In all cases, cell suspensions were blocked and labeled with a primary antibody, washed, and reacted with a goat anti-rabbit IgG secondary antibody conjugated to an Alexa Fluor probe. Cell preparations processed in parallel with primary antibody omitted were used as negative controls to set gates. Immediately before analysis, 4′,6-diamidino-2-phenylindole (DAPI) was added at a final concentration of 500 nM to detect and exclude nonviable cells [3,4,39].

For some experiments, wild-type OSCs were transfected using Lipofectamine LTX with a plasmid containing the *pDsRed2-1* expression vector under the control of a previously characterized 1.4 kb fragment of the murine *Stra8* promoter [51], and then selected using G418 to generate a stable *pStra8-DsRed*-expressing OSC line [21,55]. To avoid the use of trypsin-based dissociation solutions, which can cleave external epitopes of Ddx4 utilized for antibody-based sorting of viable OSCs [4], the cells were collected from cultures by briefly washing in 50 mM EDTA and gentle cell scraping. After centrifugation and washing, the retrieved cells were analyzed for COOH-Ddx4, CD61, SSEA1, or DsRed expression (viz. *Stra8* promoter activation) by flow cytometry [3,4,21,39].

In brief, cell samples were blocked and incubated with a 1:20 dilution of Abgent COOH-Ddx4 antibody conjugated to Alexa Fluor-594, a 1:40 dilution of a hamster anti-mouse CD61 antibody conjugated to BV786 (740867, Clone 2C9.G2; BD Biosciences, Franklin Lakes, NJ), or a 1:250 dilution of a mouse monoclonal antibody against SSEA1 conjugated to DyLight-650 (MA1022D650, Clone MC-480). After labeling, the samples were washed and analyzed using a BD FACSAria™ III (BD Biosciences) with FACSDiva software (version 10.2) [3,4,39]. To assess in vitro formation of oocyte-like cells as a sensitive bioassay to monitor OSC differentiation [3,4,6,7,10,11,13,15,21,52,56], 2.5 × 10^4^ OSCs were plated in triplicate wells of plastic 24-well tissue culture plates containing 0.5 mL of OSC culture medium for each experimental replicate, as detailed previously [3,4]. At the indicated times, 20% of the culture medium (0.1 mL) was removed and evaluated by light microscopy for in vitro-derived (IVD) oocyte formation.

### Immunolabeling of viable versus fixed and permeabilized OSCs

For immunodetection of specific regions of Ddx4 protein (carboxy vs. amino terminus), OSCs were collected from culture, rinsed in 1 × -concentrated phosphate-buffered saline (PBS), resuspended at a concentration of 1 × 10^6^ cells/mL, and prepared for extracellular protein analysis on viable cells, or intracellular protein analysis on fixed and permeabilized cells [3]. To detect externalized Ddx4 protein, viable cells were resuspended in blocking buffer containing 2% bovine serum albumin and 2% normal goat serum, and incubated on ice for 15 min. The cells were then washed with cold PBS and incubated in blocking buffer containing 5 μg/mL of the Abgent COOH-Ddx4 antibody or the NH_2_-Ddx4 antibody on ice for an additional 15 min.

After washing, all COOH-Ddx4 antibody samples were incubated on ice for 15 min in blocking buffer containing 8 μg/mL of goat anti-rabbit secondary antibody conjugated to Alexa Fluor-488, and all NH_2_-Ddx4 antibody samples were incubated on ice for 15 min in blocking buffer containing 8 μg/mL of goat anti-rabbit secondary antibody conjugated to Alexa Fluor-700. Samples incubated in blocking buffer alone (viz. no primary antibody) and processed in parallel under the same workflow with secondary antibodies served as controls. After washing in PBS, all samples were resuspended in flow cytometry buffer containing 0.1% fetal bovine serum (FBS) and 2.5 mM EDTA, and then analyzed by FACS as described above (see the Analysis of Cultured OSCs section).

For detection of intracellular Ddx4 protein, cell suspensions were prepared as described above and fixed (2% formaldehyde in PBS) for 5 min at 22°C, washed with PBS, resuspended in permeabilization buffer (0.1% Triton X-100) for 5 min at 22°C, and washed again with PBS containing 0.01% Triton X-100. Following permeabilization, the fixed samples were immunolabeled with COOH-Ddx4 antibody or NH_2_-Ddx4 antibody as outlined above. Gates were established based on control samples processed in parallel but with primary antibodies omitted, and data were collected on a BD FACSAria III using FACSDiva software, followed by analysis with FlowJo (version 8). This approach of single-protein dual-epitope analysis of Ddx4 in viable versus fixed and permeabilized OSCs has been described previously for cells freshly isolated from adult mouse ovaries [3].

### Analysis of follicle numbers in adult mouse ovaries

Ovaries collected from female mice at 3 or 20 months of age were cleaned of nonovarian tissue, fixed, sectioned, stained, and processed for histomorphometry-based quantification of the number of healthy or atretic oocyte-containing follicles at the indicated stages of development, as detailed [21].

### Analysis of OSCs in ovaries during adult life

For primary (noncultured) cell analysis, ovaries were collected and pooled from five female mice at 2, 6, 10, 15 and 20 months of age for each experimental replicate, which was then repeated at least three times. The ovaries were cleaned of any surrounding nonovarian tissue, dissociated, and prepared for flow cytometry (see the Analysis of Cultured OSCs section) or for gene expression (see the Gene Expression Analysis section).

### ALDH activity assays

To measure the relative levels of ALDH activity in OSCs, we used the ALDEFLUOR™ kit following the manufacturer's guidelines for flow cytometry-based assessment in viable cells (STEMCELL Technologies, Cambridge, MA). Briefly, cultured wild-type or *pStra8-DsRed* OSCs were brought to a concentration of 1 × 10^6^ viable cells in 1 mL of ALDEFLUOR assay buffer. After addition of 5 μL of activated ALDEFLUOR reagent, samples were incubated for 45 min at 4°C under 5% CO_2_-95% air. For control samples, the ALDH inhibitor, diethylaminobenzaldehyde (DEAB) [57], was added at a final concentration of 15 μM immediately following the addition of activated ALDEFLUOR reagent. Following incubation, the samples were centrifuged, resuspended in assay buffer, and analyzed by flow cytometry with DEAB-treated samples used to set the gates for identification of cells with high ALDH activity (“Aldefluor-Bright”) versus those cells with low to no detectable levels of ALDH activity (“Aldefluor-Dim”).

For some experiments, samples were maintained on ice for immunolabeling using a 1:250 dilution of SSEA1 antibody before analysis by flow cytometry. In other experiments, OSC cultures were pretreated for 24 h without (vehicle control) or with 10 nM E2 to induce OSC differentiation [52] before ALDH activity analysis.

### PKH67 label retention assays

To assess relative quiescence, *pStra8-DsRed* OSCs were labeled with the green-fluorescent aliphatic dye, PKH67, according to the manufacturer's protocol (MilliporeSigma, Burlington, MA). With each cell division, the degree of total label retention, measured by mean fluorescence intensity (MFI), is reduced by ∼50% in each daughter cell compared with that in the parent cell or in nondividing cells. Identification of stem cells by these types of label retention approaches has been used for decades across cell types [58–62]. To do this, single-cell suspensions were washed in serum-free culture medium and resuspended to a final concentration of 1 × 10^7^ cells/mL before the addition of PKH67 at a final concentration of 2 × 10^−6^ M. The cells were then incubated for 5 min at 22°C before neutralizing the reaction by dilution (1:1, vol:vol) with FBS. Cells were then washed and resuspended in flow cytometry buffer to sort labeled cells, which were returned to culture for 5 days to allow for expansion. The cells were then collected and analyzed for PKH67 MFI (label retention) and for SSEA1 or DsRed expression (viz. *Stra8* promoter activation).

### Gene expression analysis

Total RNA was collected from cultured OSCs using RNAzol RT (Molecular Research Center, Cincinnati, OH), treated with RNase-free DNase-I, and reverse transcribed into complementary DNA (cDNA) using RevertAid. Conventional polymerase chain reaction (PCR) analysis was conducted as detailed [3], using primers detailed in Supplementary Table S1. For quantitative PCR, gene expression analysis was performed using the primers listed in Supplementary Table S2 along with GoTaq Green Master Mix (Promega, Madison, WI), following the manufacturer's protocol. Expression analysis was done with the ΔΔC_t_ method of relative quantitation using SYBR Green and *beta-2-microglobulin* as the sample reference gene [10].

### Human ovary snRNA-seq data analysis

We accessed data sets for comparative snRNA-seq analysis of ovarian tissue biopsies collected from four women at 23, 27, 28, and 29 years of age (young adult) versus four women at 49, 51, 52, and 54 years of age (peri/postmenopausal), which were deposited by Jin et al. [53] (available through National Center for Biotechnology Information Gene Expression Omnibus Accession No. GSE202601; https://www.ncbi.nlm.nih.gov/geo/query/acc.cgi?acc=GSE202601); all tissues were flash-frozen autopsy samples of sudden death individuals with normal ovarian histology. After preprocessing of the data using 10xGenomics Cell Ranger 7.0.0 software, further filtering and analysis were performed in Seurat using R-Studio (for quality control) and lines of code we have published previously [14] (https://github.com/hanrico/Ovarian-scRNA-seq). For quality control, genes expressed in no <3 nuclei were retained for analysis, and nuclei expressing 200–6,000 genes, with no more than 15% of these genes being mitochondrial in nature, were included in the downstream analyses.

To select these parameters, violin plots and scatter plots depicting mitochondrial gene percentage, “nFeature-RNA,” and “nCount-RNA” were prepared and visualized in Seurat [14].

Once filtering was completed, the remaining nuclei from each sample within each age group were integrated using the canonical correlation analysis integration tool in Seurat. After successful integration, the preprocessed data were merged and regressed for batch effects. Column normalization and log transformation were conducted before principal component analysis. To further analyze the data generated by Cell Ranger 7.0.0, elbow plots were generated in Seurat, which assisted in the determination of the ideal number of principal components to include in downstream Uniform Manifold Approximation and Projection (UMAP) dimensional reduction. To maintain consistency with the analysis completed by Jin et al. [53], UMAP projections were prepared with the first 15 principal components and a setting of 0.1 for resolution. For gene expression analysis in 10xGenomics Loupe Browser, data for the four samples from each age group were aggregated together using Cell Ranger Aggregate.

Following aggregation of the young adult age group data, the 24,593 nuclei maintained in the output file were reduced to 24,068 nuclei for Loupe Browser; following aggregation of the peri-/postmenopausal age group data, the 20,096 nuclei maintained in the Cell Ranger Aggregate file were reduced to 19,853 nuclei for Loupe Browser. The following parameters were used for quality filtering of aggregated data for Loupe Browser: minimum thresholds for features expressed, 200; maximum thresholds for features expressed, 6,000; maximum mitochondrial unique molecular identified counts, 15%; number of principal components, 15; minimum distance, 0.2; number of neighbors, 8.

### Data presentation and analysis

All experiments with mice were independently repeated at least three times (see legends to Figs. 2–8 for details). Representative images are shown for qualitative analysis, where graphs represent the mean ± standard error of the mean of combined data from the replicate experiments. Data analysis was performed using analysis of variance followed by Student's *t*-test to determine statistical significance.

## Results

### Epitope mapping of Ddx4 termini in cultured OSCs of mice

**FIG. 1.**
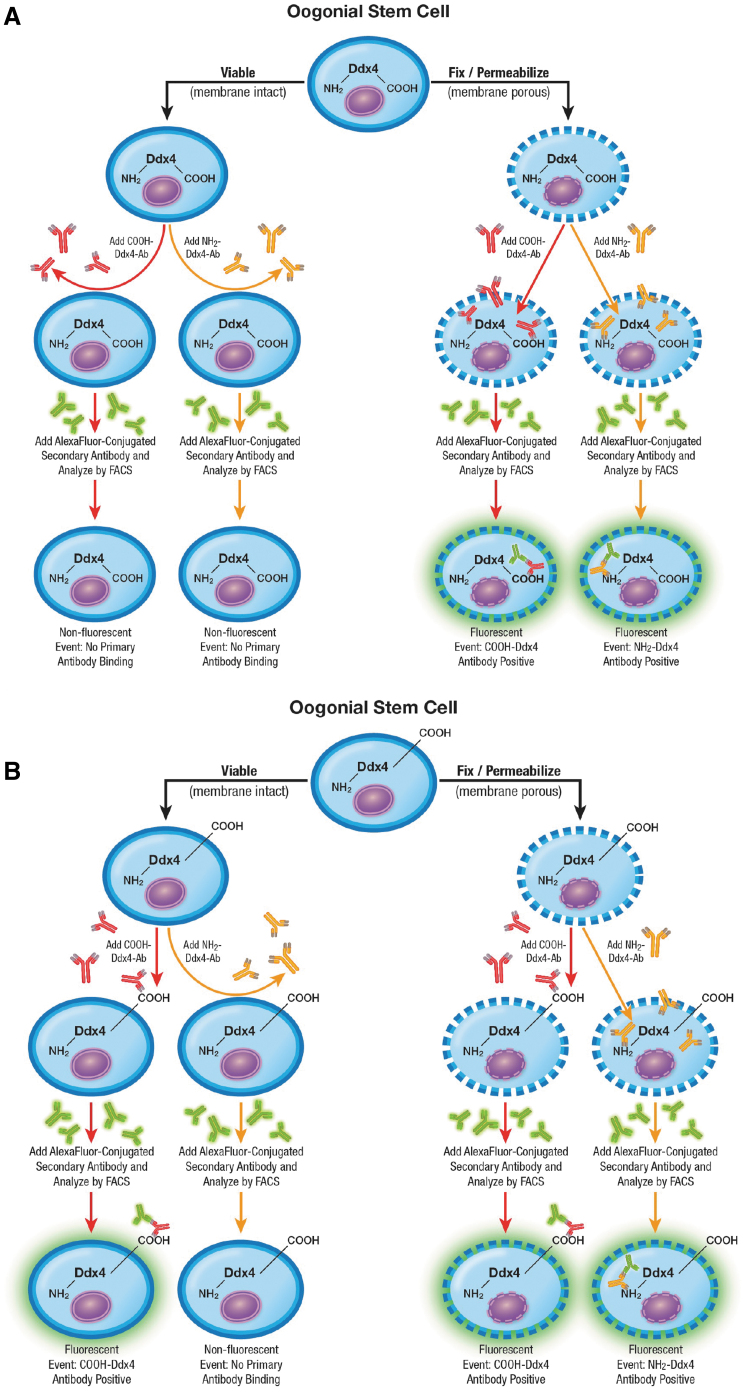
Schematic depiction of predicted Ab-based epitope mapping outcomes using fluorescence activated cell sorting if Ddx4 is completely cytoplasmic **(**internalized; **A)** or if the carboxy terminus of the protein is exposed on the outer cell surface of viable cells **(B)**. Refer to Fig. 2 for outcomes of the experiments designed to test which of these models is correct, using COOH-Ddx4 antibodies (COOH-Ddx4-Ab) and NH_2_-Ddx4 antibodies (NH_2_-Ddx4-Ab) with mouse OSCs as sample input for flow cytometry. Ab, antibody; COOH-Ddx4, carboxy terminus of DEAD box helicase-4; NH_2_-Ddx4, amino terminus of DEAD box helicase-4; OSC, oogonial stem cell.

To verify that the carboxy terminus of Ddx4 is exposed on the outer surface of OSCs [3,4,16], we conducted a dual-epitope analysis of Ddx4 in cultured OSCs to clearly define the localization patterns of the carboxy and amino termini of the protein using antibodies against COOH-Ddx4 and NH_2_-Ddx4, respectively. If Ddx4 is entirely cytoplasmic, the protein should only be detectable in OSCs, by either antibody, if the cells are first fixed and permeabilized before the analysis (Fig. 1A).

**FIG. 2.**
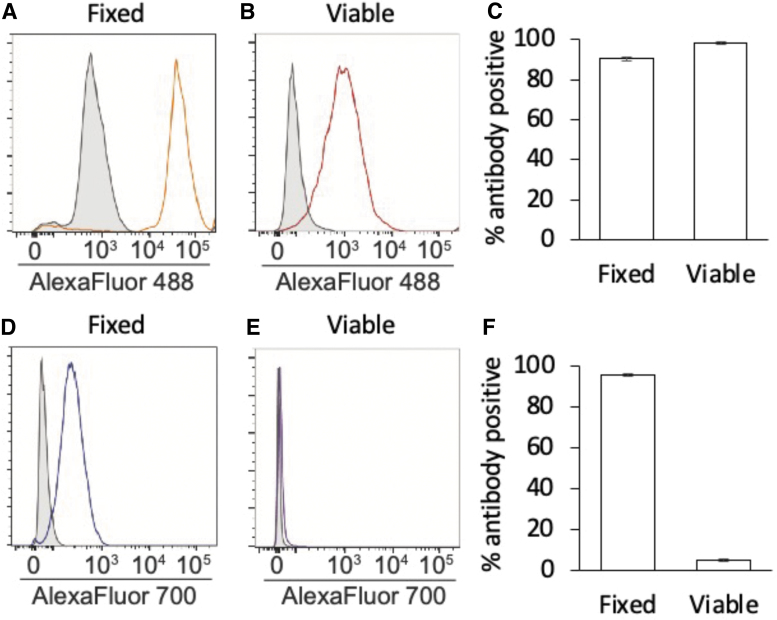
Flow cytometric antibody-based mapping of the COOH and NH_2_ termini of Ddx4 in cultured mouse OSCs. After gating against Alexa Fluor 488-conjugated secondary antibody-only controls **(***gray-shaded peaks* in the histogram plots shown in **A** and **B)**, near-complete primary antibody-positive fluorescence shifts **(***open peaks* in the histogram plots shown in **A** and **B)** were obtained when antibodies that target COOH-Ddx4 were used, irrespective if the cells were fixed and permeabilized **(A, C)** or nonfixed and nonpermeabilized **(**viable: **B**, **C)** at the time of analysis. However, when NH_2_-Ddx4 antibodies were used, a near-complete primary antibody-positive fluorescence shift **(**compare the *open peaks* against the *gray-shaded peaks*, the latter representing Alexa Fluor 700-conjugated secondary antibody-only controls, in the histogram plots shown in **D** and **E)** was only obtained if the cells were fixed and permeabilized for the analysis **(D, F)**. In contrast, if the cells were viable (nonfixed and nonpermeabilized) at the time of the analysis using NH_2_-Ddx4 antibodies, only 5% of the events were identified as showing a slightly higher fluorescence intensity over secondary antibody-only controls **(F)**, but this did not produce a discernible fluorescence intensity shift **(E**, note the absence of a second peak distinct from the secondary antibody-only controls; compare with data shown in **A**, **B**, and **D)**. Results are the mean ± SEM, *n* = 3 independent experiments. NH_2_-Ddx4, amino terminus of Ddx4; OSC, oogonial stem cell; SEM, standard error of the mean.

Conversely, if the carboxy terminus of Ddx4 is exposed on the outer surface of the cells to permit viable cell sorting [3,4,16,39], COOH-Ddx4 antibodies should identify positive cells irrespective of whether the cells are fixed and permeabilized for the analysis since the epitope is exposed on viable cells; however, NH_2_-Ddx4 antibodies should still identify positive cells only if the cells are fixed and permeabilized beforehand to enable the antibody to bind with its intracellular target (Fig. 1B).

Gating against Alexa Fluor 488-conjugated secondary antibody-only controls, a nearly two-log-order shift in fluorescence intensity was detected when antibodies against the carboxy terminus of Ddx4 were used with samples that were either fixed and permeabilized (90.47% ± 0.99% primary antibody-positive events) or nonfixed and nonpermeabilized (viable; 98.26% ± 0.92% primary antibody-positive events) for the analysis (Fig. 2A–C). When antibodies against the NH_2_-Ddx4 were used, a comparable two-log-order shift in fluorescence intensity was also observed if the cells were fixed and permeabilized for the analysis (95.60% ± 0.60% primary antibody-positive events) (Fig. 2D, F).

In stark contrast, no discernible shift in fluorescence intensity versus secondary antibody-only controls was detected when viable (nonfixed and nonpermeabilized) cells were analyzed with the NH_2_-Ddx4 antibody (Fig. 2E). Moreover, the 5.10% ± 0.60% events identified as “NH_2_-Ddx4 antibody-positive” in viable cell samples (Fig. 2F) showed no clear shift in fluorescence intensity versus secondary antibody-only controls (Fig. 2E), suggesting that few if any cells were recognized by the NH_2_-Ddx4 antibody.

These findings not only agree with prior Ddx4 epitope mapping studies of freshly isolated OSCs [3,16], but also verify that the vast majority of the cells established in culture following their isolation from adult mouse ovaries using FACS and COOH-Ddx4 antibodies remain positive for Ddx4 irrespective of whether the same (COOH-Ddx4) or a different (NH_2_-Ddx4) antigenic region is used to identify the protein. Our experimental design here also included a second level of antibody verification in that the cells established in culture were initially isolated from adult mouse ovaries using a COOH-Ddx4 antibody from Abcam, whereas our subsequent epitope mapping studies of these cells were conducted with a different COOH-Ddx4 antibody from Abgent. This approach was taken to rule out the possibility of any potential artifact associated with use of the same COOH-Ddx4 antibody for both isolation of the cells and downstream analysis of the same cells once established in culture.

### Changes in OSCs across adult life span in mice

**FIG. 3.**
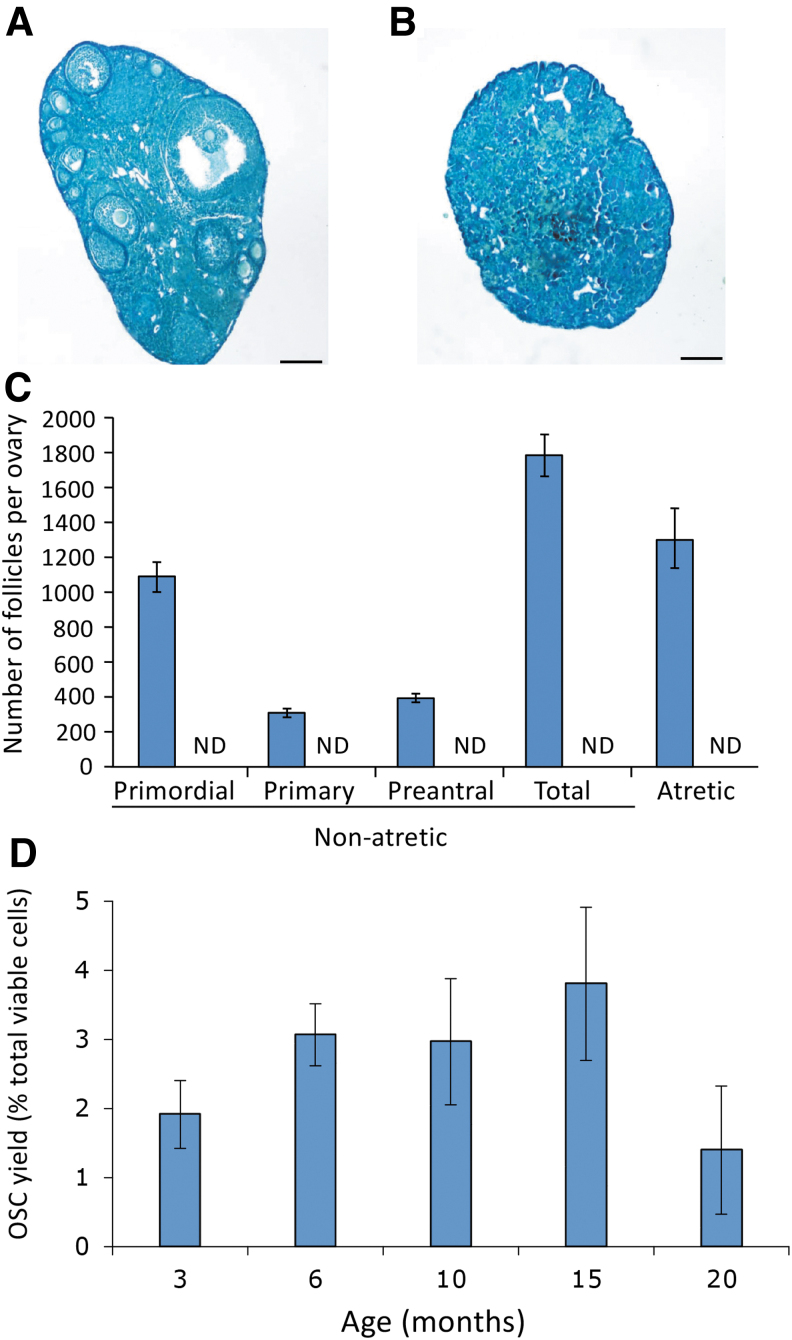
Analysis of mouse OSCs across life span. Comparative analysis of mouse ovaries at 3 versus 20 months of age based on histological appearance [**(A)** 3 months of age; **(B)** 20 months of age; scale bars, 50 μm] and oocyte-containing follicle numbers [**(C)** *blue bars* designate follicle counts per ovary at 3 months of age, whereas no follicles at any stage of development were detected in ovaries of mice at 20 months of age: ND]. Results are the mean ± SEM with five mice analyzed per age group. Age-related changes in viable OSC yield per ovary, expressed as a percent of total viable cells sorted by flow cytometry using COOH-Ddx4 antibodies **(D)** Results are the mean ± SEM, *n* = 3 with ovaries from four to six mice of each age pooled for each experimental replicate. ND, nondetectable; OSC, oogonial stem cell; SEM, standard error of the mean.

**FIG. 4.**
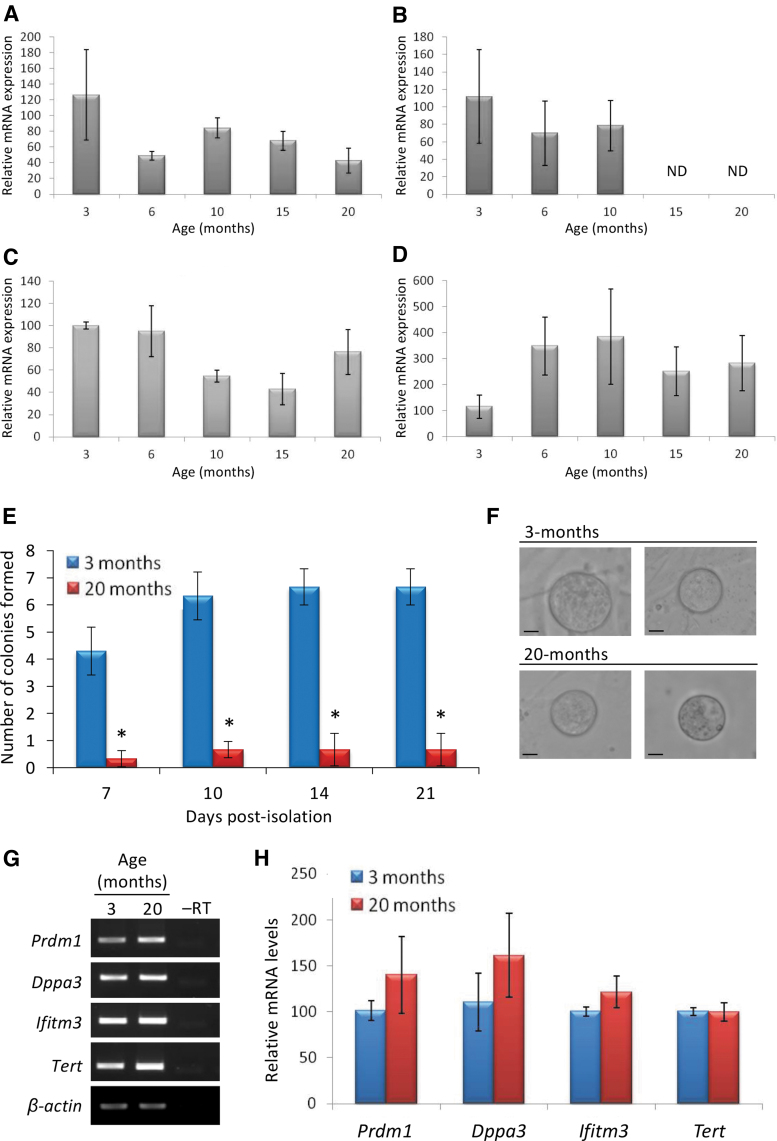
Primitive germ line gene expression patterns in mouse OSCs throughout adulthood. Quantitative PCR analysis of *Prdm1* **(A)**, *Dppa3* **(B)**, *Ifitm3* **(C)**, and *Tert* **(D)** expression in freshly isolated OSCs collected from female mice at the indicated ages. Results are the mean ± SEM with ovaries from four to six mice of each age pooled for each experimental replicate, which was then repeated three to five times. Comparison of colony formation rates over the first 3 weeks of in vitro culture after plating equivalent numbers of OSCs (1 × 10^2^) isolated from ovaries of young versus aged mice **(E)**. Results are the mean ± SEM, *n* = 3 independent experiments (*P < 0.05). Examples of IVD oocytes generated by OSCs isolated from mice at 3 and 20 months of age after establishment in culture (scale bars, 10 μm) **(F)** Conventional **(G**; *β-actin*, housekeeping gene used as an RNA sample loading control**)** and quantitative **(H)** PCR analyses of primitive germ line gene expression in OSCs isolated from 20-month-old female mice and cultured in vitro **(**compare with freshly isolated OSCs at 20 months of age in **A–D)**. Results are the mean ± SEM, *n* = 3 independent experiments. *Dppa3*, *developmental pluripotency associated 3*; *Ifitm3*, *interferon-induced transmembrane protein 3*; IVD, in vitro derived; ND, nondetectable; OSC, oogonial stem cell; PCR, polymerase chain reaction; *Prdm1*, *PR domain zinc finger protein 1*; SEM, standard error of the mean; *Tert*, *telomerase reverse transcriptase*.

From early adulthood (3 months) to postreproductive advanced age (20 months), OSCs could be retrieved from ovaries, even after the oocyte-containing follicle pool had been completely depleted (Fig. 3A–D). Expression profiling of freshly isolated (noncultured) OSCs retrieved from the ovaries of female mice at 3 versus 20 months of age revealed that of the four genes analyzed that are critical to the function of primitive germ cells [*PR domain zinc finger protein 1* or *Prdm1*; *developmental pluripotency associated 3* or *Dppa3*; *interferon-induced transmembrane protein 3* or *Ifitm3*; *telomerase reverse transcriptase* or *Tert*] [63–65], only *Dppa3* expression was lost with age (Fig. 4A–D).

Although colony formation was lower in cultures established from OSCs isolated at 20 versus 3 months of age (Fig. 4E), in vitro oogenesis was detected in both sets of cultures (Fig. 4F), and this occurred in parallel with a return of *Dppa3* expression in cultured OSCs initially isolated at 20 months of age (Fig. 4G, H; compare with Fig. 4B).

### Immunophenotyping of OSC subpopulations in mice

**FIG. 5.**
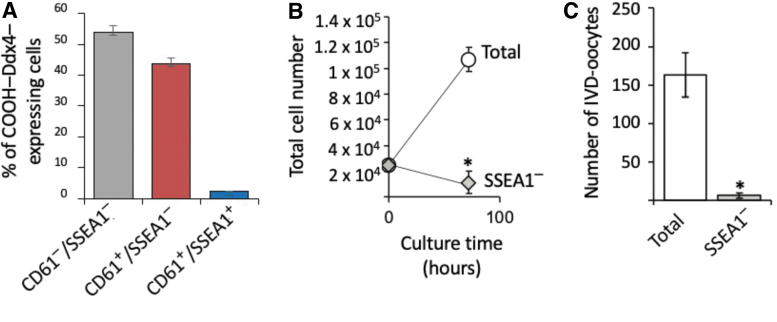
Identification of mouse OSC subpopulations. Subpopulation distribution analysis of cultured OSCs identified by FACS as positive for externalized COOH-Ddx4 (98.26% ± 0.92% of the total cells) that are negative for CD61 and SSEA1 (CD61^−^/SSEA1^−^) that coexpress CD61 but not SSEA1 (CD61^+^/SSEA1^−^), or that coexpress both CD61 and SSEA1 (CD61^+^/SSEA1^+^), noting that the rare SSEA1-expressing subpopulation represents only 2.25% ± 0.14% of the total COOH-Ddx4^+^ cell population **(A)**. Results are the mean ± SEM, *n* = 3 independent experiments. Analysis of total cell numbers **(B)** and IVD-oocyte formation rates **(C)** in OSC cultures without (control; Total) or with (SSEA1^−^) immunodepletion of the SSEA1-expressing OSC subpopulation before passage and culture for 72 h. Results are the mean ± SEM, *n* = 3 independent experiments (*P < 0.05). CD61, cluster of differentiation 61; IVD, in vitro derived; OSC, oogonial stem cell; SEM, standard error of the mean; SSEA1, stage-specific embryonic antigen 1.

To gain initial insights into potential heterogeneity within OSCs, the cells were isolated from young adult mouse ovaries using COOH-Ddx4 antibody-based sorting and established as actively dividing cultures for immunophenotyping analysis. In cultured OSCs simultaneously labeled with antibodies against COOH-Ddx4, SSEA1, and CD61 (Fig. 5A), we observed that 98% ± 1% of the viable cells were identified as positive for COOH-Ddx4. Further analysis indicated that 46% of the COOH-Ddx4-positive cells also expressed CD61, but only 2% of the cells were identified as positive for all three cell surface markers (Fig. 5A).

To explore the potential importance of the rare SSEA1-expressing cells to OSC culture dynamics, we used SSEA1-specific antibodies with flow cytometry to deplete the SSEA1-expressing subpopulation before replating of the cells following passage. Three days after replating, we observed that cell numbers and IVD oocyte formation rates were both significantly reduced in SSEA1-depleted OSC cultures when compared with nondepleted (total) OSC cultures (Fig. 5B, C).

### Immunophenotyping of OSC subpopulations in young and aged mice

**FIG. 6.**
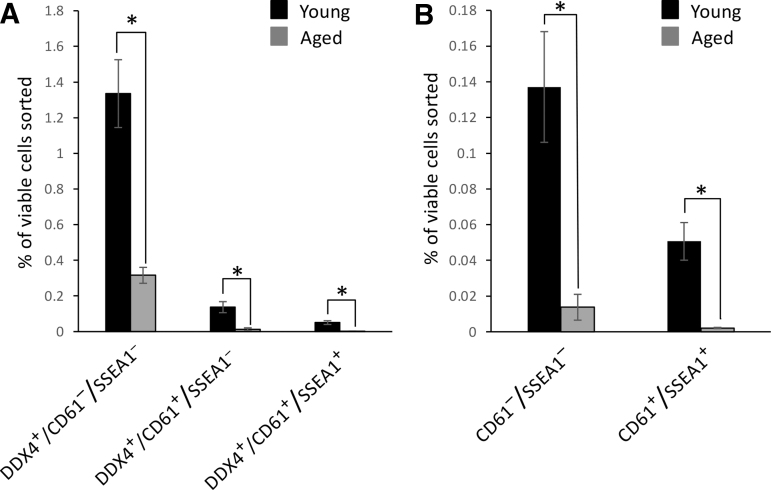
Analysis of OSC subpopulations in ovaries of young and aged mice. Subpopulation distribution analysis of OSCs freshly isolated from mice at 2–3 months of age (young) or 20 months of age (aged) and identified by FACS as positive for COOH-Ddx4 only (COOH-Ddx4^+^/CD61^−^/SSEA1^−^), double-positive for COOH-Ddx4 and CD61 (COOH-Ddx4^+^/CD61^+^/SSEA1^−^), or triple-positive for COOH-Ddx4, CD61, and SSEA1 (COOH-Ddx4^+^/CD61^+^/SSEA1^+^) **(A)**. Note that data corresponding to analysis of the COOH-Ddx4^+^/CD61^+^/SSEA1^−^ and COOH-Ddx4^+^/CD61^+^/SSEA1^+^ subpopulations shown in **(A)** are enlarged for visual clarity in **(B).** Results are the mean ± SEM, *n* = 3 independent experiments with ovaries pooled from three to six mice for each experimental replicate (*P < 0.05). CD61, cluster of differentiation 61; OSC, oogonial stem cell; SEM, standard error of the mean; SSEA1, stage-specific embryonic antigen 1.

To next evaluate if similar OSC subpopulations exist in vivo, ovarian tissue was collected from female mice at 2–3 months of age (young) or 20 months of age (aged) and dissociated for FACS-based analysis of COOH-Ddx4, CD61, and SSEA1 expression. This analysis revealed that while the yields of COOH-Ddx4–positive, COOH-Ddx4/CD61–double-positive, and COOH-Ddx4/CD61/SSEA1–triple-positive cells all declined with age, all three subpopulations remained detectable in the ovaries of aged female mice at close to the same ratios as those observed in young adult mouse ovaries (Fig. 6).

### Analysis of OSCs based on measures of quiescence versus differentiation

**FIG. 7.**
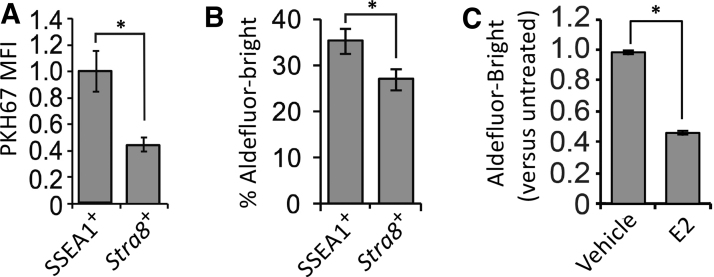
Deeper analysis of the SSEA1-expressing OSC subpopulation. Relationship between PKH67 MFI (label retention) and SSEA1 expression or expression *(Stra8)* in cultured mouse OSCs 5 days after aliphatic dye labeling **(A).** Comparative assessment of the (SSEA1^+^) frequency of Aldefluor-Bright OSCs in SSEA1-expressing versus *Stra8*-expressing cell populations **(B)**. Change in the frequency of Aldefluor-Bright OSCs following 24 h of culture with vehicle or 10 nM E2 relative to control OSC cultures before treatment **(C)**. All results are the mean ± SEM, *n* = 3 independent experiments (*P < 0.05). E2, estradiol; MFI, mean fluorescence intensity; OSC, oogonial stem cell; SEM, standard error of the mean; SSEA1, stage-specific embryonic antigen 1; *Stra8*, *stimulated by retinoic acid gene 8*.

Past studies with mice have demonstrated that SSEA1 is highly expressed in PGCs during embryogenesis [49], and that antibodies against SSEA1 can be used to sort PGCs from fetal gonads [66] and PGCLCs from differentiating pluripotent stem cell cultures [38]. To explore if SSEA1-expressing OSCs represent a primitive PGC-/PGCLC-like subpopulation, we evaluated the relationship between SSEA1 expression, PKH67 label retention, ALDH activity, and *Stra8* promoter activation. In cultured OSCs 5 days after aliphatic dye labeling, we observed that SSEA1-expressing OSCs maintained fluorescence intensity indicative of nearly complete PKH67 label retention (viz. quiescence) during culture, whereas PKH67 fluorescence intensity was reduced by ∼50% in *Stra8*-expressing OSCs after 5 days of culture (Fig. 7A). In addition, a significantly higher percentage of Aldefluor-Bright OSCs were identified as SSEA1-expressing cells when compared with *Stra8*-expressing cells (Fig. 7B).

Our interpretation of these findings that SSEA1-expressing cells represent a more primitive or quiescent OSC subpopulation was further tested by treating OSCs with E2, which drives meiotic differentiation of the cells in vitro and in adult ovaries in vivo through estrogen receptor alpha (ERα)-mediated transcriptional activation of *Stra8* [52]. In support of the existence of an inverse relationship between ALDH activity and differentiation, 24 h of treatment with E2 significantly decreased the percentage of Aldefluor-Bright OSCs in the cultures by nearly 50% (Fig. 7C).

### Analysis of human ovarian aging snRNA-seq data

**FIG. 8.**
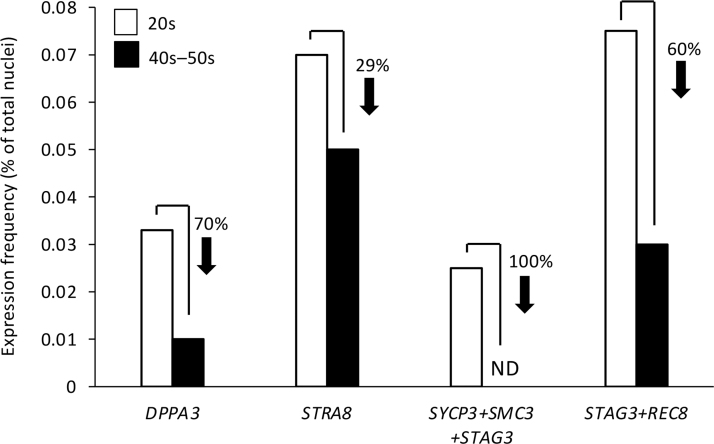
Analysis of human ovarian aging snRNA-seq data. Frequency of nuclei with expression of *DPPA3* or *STRA8*, coexpression of core meiotic recombination genes (*SYCP3*+*SMC3*+*STAG3*), or co-expression of *STAG3* and *REC8* in ovarian tissue samples collected from women in their 20s (young adult) versus late 40s to early 50s (peri-/postmenopausal). The analyses were performed on aggregated data from snRNA-seq analysis of four independent biopsy samples per age group. *DPPA3*, *developmental pluripotency associated 3*; ND, none detected; *SMC3*, *structural maintenance of chromosomes 3*; snRNA-seq, single-nucleus RNA sequence; *STAG3*, *stromal antigen 3*; *STRA8*, *stimulated by retinoic acid gene 8*; *SYCP3*, *synaptonemal complex protein 3*.

In a final experiment, we analyzed snRNA-seq data sets derived from ovarian biopsies of young adult versus peri-/postmenopausal women for clues of conserved features of ovarian aging between mice and humans. Based on the data obtained from our studies of mouse ovaries (Fig. 4B), we first focused on *DPPA3* and observed that expression frequency of this key germ cell differentiation gene was reduced by 70% in reproductively aged versus young adult ovarian tissue (Fig. 8). Nuclear expression frequency of the early meiotic commitment gene *STRA8*, which is a target of *DPPA3*-mediated regulation in OSCs [67], was reduced by 29% in reproductively aged ovarian tissue (Fig. 8).

In alignment with these observations, which point to a negative impact of aging on the expression of genes and pathways that support de novo oogenesis, nuclei with coexpression of the three critical genes required for meiotic recombination and early meiotic progression in germ cells—*synaptonemal complex protein 3* (*SYCP3*), *structural maintenance of chromosomes 3* (*SMC3*) and *stromal antigen 3* (*STAG3*) [68–73], while detectable in young adult ovaries at a frequency of around 0.025%, were absent in reproductively aged ovarian tissue (Fig. 8). We also observed that the frequency of nuclei with coexpression of *STAG3* and the meiosis-specific kleisin family member, *REC8* [74], was reduced by 60% in peri-/postmenopausal ovarian tissue samples compared with young adult ovarian tissue samples (Fig. 8).

## Discussion

Following the first isolation of OSCs from adult mouse ovaries in 2009 [16], the majority of the more than 80 articles published on these cells in diverse species over the years have relied on cell-sorting strategies that involve antibody-based detection of an externalized epitope of Ddx4 to obtain viable OSCs for downstream characterization and use (reviewed in [2]; see also supporting information table 1 in Alberico et al. [14] for a more complete literature overview).

While other cell surface markers of primitive germ cells, such as Ifitm3 (also referred to as Fragilis), have been used alone or in combination with Ddx4 antibodies to successfully isolate OSCs (eg, see Sequeira et al. [11] and Navaroli et al. [39]; see also supporting information table 2 in Alberico et al. [14] for a more complete listing of published studies using Ifitm3 antibody-based sorting of OSCs), a hallmark feature of these cells across many studies is the externalization of the carboxy terminus of Ddx4. However, as OSCs commit to, and differentiate through, meiosis, the carboxy terminus of Ddx4 is progressively lost from the cell surface [55], with complete internalization to become a cytoplasmic protein in oocytes [3].

Over a decade ago, the specificity of detecting COOH-Ddx4 on the surface of freshly isolated OSCs was established by cell imaging and single-protein dual-epitope antibody mapping [3,16]. In this study, we have verified, using antibodies that target the carboxy versus amino terminus of Ddx4, that the carboxy terminus of the protein remains exposed on the surface of viable OSCs after establishment of the cells in vitro. Furthermore, the cells continue to express Ddx4 protein in culture, which was confirmed through the use of two different antibodies against different antigenic regions of the protein.

These observations once again highlight the discordance in questions raised by some scientists about the validity of COOH-Ddx4 antibody-based sorting to isolate OSCs [40–42] (see also Woods and Tilly [43], Alberico et al. [14], and Woods and Tilly [44]) with what has been experimentally established, and reproduced, by multiple laboratories since 2009 (see supporting information table 1 in Alberico et al. [14] for a more comprehensive listing of published studies using COOH-Ddx4 antibody-based sorting approaches to study OSCs across species). In this regard, related ongoing claims of an absence of evidence supporting the existence of functional OSCs in adult ovaries [42]—with function defined by the ability of OSCs to differentiate into new oocytes that mature into competent eggs that can be fertilized to produce embryos and viable offspring, are equally discordant with the numerous published studies that have unequivocally established the functionality of OSCs through multiple approaches (reviewed in Woods and Tilly [44]).

These include intraovarian transplantation of genetically traceable OSCs into wild-type female mice to map the production of donor OSC-derived offspring [3,16–22], in vivo germ line lineage tracing to the point of offspring generation [21], and in vitro production of competent eggs that generate offspring following guided differentiation of mouse OSCs using three-dimensional ovarian organoids [23]. Notably, these latter studies used the same approaches as those used, and widely accepted, for the generation of functional female gametes from pluripotent stem cells in mice for over 10 years [38,75–77].

Thus, while a large body of published data collectively support that functional OSCs exist in mammals, it is unknown if the cells retrieved from dispersed ovarian tissue using COOH-Ddx4 antibodies represent a relatively homogenous pool of similar cells or, conversely, are comprised of a heterogenous population of female germ line stem and progenitor cells that differ phenotypically and functionally. In their studies of human ovarian tissue, Clarkson et al. [9] provided evidence that ALDH activity levels vary in isolated OSCs, suggesting that the cell population as a whole is not homogenous. In this study, we have verified and extended these observations by showing that mouse OSCs differ not only in the levels of ALDH activity but also in the expression of two cell surface markers of primitive germ cells—CD61 and SSEA1.

We also found that these subpopulations, defined by cell surface marker profiles, exhibit differences in their degree of differentiation. For the latter, we observed that transcriptional activation of the *Stra8* gene, which is considered one of the earliest steps in the commitment of germ cells to meiosis [50,51], was inversely associated with both ALDH activity levels and SSEA1 expression. Conversely, OSC cultures treated with E2, which enhances the meiotic differentiation of these cells into oocytes [52], reduced the percentage of Aldefluor-Bright cells, consistent with higher numbers of OSCs transitioning from a primitive to more differentiated state in response to E2. Importantly, this cellular heterogeneity was not an artifact of in vitro culture in that COOH-Ddx4-positive, COOH-Ddx4/CD61–double-positive, and COOH-Ddx4/CD61/SSEA1–triple-positive subpopulations were also identified in ovaries, irrespective of the age at the time of tissue collection.

Another important observation from our studies arose from the evaluation of gene expression in freshly isolated mouse OSCs over the course of female life span, which uncovered a loss in the expression of specifically *Dppa3* in OSCs between 10 to 15 months of age. This time period in mice is interesting in that it represents the transition from declining reproductive performance (10 months of age) to complete oocyte exhaustion and reproductive failure (15 months of age) [78–80]. Prior studies using inducible suicide gene technology targeted to primitive germ cells during meiotic commitment have established that female mice lose the capacity to maintain de novo oogenesis around 10 months of age, due to a defect in the ability of OSCs to progress through meiosis [21].

These data, when considered with our observations on the loss of *Dppa3* expression in OSCs with age and with recent data indicating that Dppa3 acts as an epigenetic enhancer of meiotic commitment in mouse OSCs [67], implicate Dppa3 as a key player in aging associated OSC dysfunction. In further support of this conclusion, we found that OSCs isolated from mouse ovaries long after complete depletion of the oocyte pool (20 months of age) retained the capacity to generate IVD oocytes in culture and this was associated with a reactivation of *Dppa3* expression in the cells.

Considering these data, we were intrigued by the outcomes of our targeted analysis of snRNA-seq data sets derived from ovarian biopsies of young adult versus peri-/postmenopausal women, which revealed that the expression frequency of *DPPA3* in reproductively aged ovaries was only 30% of that detected in young adult ovaries. Consistent with a key role for DPPA3 in promoting the meiotic differentiation of primitive germ cells [67], our analysis also showed that this decline in *DPPA3* expression with age occurred in parallel with a loss in coexpression frequency of three core genes required for meiotic recombination: *SYCP3*, *SMC3*, and *STAG3* [68–73]. We were particularly struck by the decrease in *STAG3* expression frequency with age since mutations in this gene in women have been directly tied to primary ovarian insufficiency (POI) [81–83].

Notably as well, cohesion of sister chromatids during meiotic progression also requires REC8, a meiosis-specific kleisin family member and binding partner for synaptonemal complex proteins [74,84,85]. As is the case with *STAG3*, *REC8* mutations have also been identified as a cause of POI in women [86,87]. Our assessment of the human ovarian snRNA-seq data sets for nuclear coexpression frequencies of *STAG3* and *REC8* revealed a 60% decline in paired expression of these genes from young adult life to peri-/postmenopausal age. Collectively, these data offer evidence of the occurrence of active meiosis in adult human ovaries under normal physiological conditions, which is consistent with results from prior studies in mice [21] and women [14]. These findings also highlight what appears to be a negative impact of maternal aging on the expression of a spectrum of genes involved in coordinating meiotic progression and oogenesis.

As interesting as these latter observations are to consider in the context of ovarian aging, we note that caution should be exercised when building conclusions based solely on changes in gene expression patterns, especially those derived from large data sets such as snRNA-seq. These types of approaches offer clues as to what may be occurring at the cellular level, but the data are still correlative at best [88]. This limitation is compounded by the fact that single-cell analytical strategies introduce a number of additional caveats as well when patterns of gene expression are used as the sole basis of conclusions drawn.

For example, in a carefully controlled assessment of the impact of flow cytometry on gene expression, it was found that FACS, per se, has a minimal transcriptomic effect in cells; however, the same study found that the enzymatic and mechanical disaggregation of the starting tissue into a single-cell suspension required for flow cytometry, and by inference snRNA-seq or scRNA-seq, triggers an upregulation in expression of numerous microRNAs, which in turn is associated with a decrease in expression of their predicted target genes [89]. Studies such as these underscore a major limitation of single-cell gene expression technologies: conclusions drawn about cell lineage identification or function based solely on transcriptomic changes across the samples being compared are not only rooted in correlation [44,88] but also can be artifactually influenced by sample preparation protocols [89].

In summary, the data presented herein support the existence of OSC subpopulations in adult mammalian ovaries, which differ in gene expression patterns and degree of differentiation commitment. Furthermore, our assessment of OSCs and oogenesis in the context of mouse and human female reproductive aging has provided evidence of marked changes in expression of the epigenetic modifier of meiotic commitment, *Dppa3*/*DPPA3* [67], which may contribute to OSC quiescence and oogenic failure in adult mammalian ovaries with advancing age [21].

## Supplementary Material

Supplemental data

Supplemental data

## References

[1] Johnson J, J Canning, T Kaneko, JK Pru and JL Tilly. (2004). Germline stem cells and follicular renewal in the postnatal mammalian ovary. Nature 428:145–150. 15014492 10.1038/nature02316

[2] Martin JJ, DC Woods and JL Tilly. (2019). Implications and current limitations of oogenesis from female germline or oogonial stem cells in adult mammalian ovaries. Cells 8:93. 30696098 10.3390/cells8020093 PMC6407002

[3] White YAR, DC Woods, Y Takai, O Ishihara, H Seki and JL Tilly. (2012). Oocyte formation by mitotically active germ cells purified from ovaries of reproductive-age women. Nat Med 18:413–421. 22366948 10.1038/nm.2669 PMC3296965

[4] Woods DC and JL Tilly. (2013). Isolation, characterization and propagation of mitotically active germ cells from adult mouse and human ovaries. Nat Protoc 8:966–988. 23598447 10.1038/nprot.2013.047 PMC5545930

[5] Woods DC and JL Tilly. (2015). Autologous germline mitochondrial energy transfer (AUGMENT) in assisted human reproduction. Semin Reprod Med 33:410–421. 26574741 10.1055/s-0035-1567826 PMC5545901

[6] Ding X, G Liu, B Xu, C Wu, N Hui, X Ni, J Wang, M Du, X Teng and J Wu. (2016). Human GV oocytes generated by mitotically active germ cells obtained from follicular aspirates. Sci Rep 6:28218. 27357640 10.1038/srep28218 PMC4928061

[7] Silvestris E, P Cafforio, S D'Oronzo, C Felici, F Silvestris and G Loverro. (2018). In vitro differentiation of human oocyte-like cells from oogonial stem cells: single-cell isolation and molecular characterization. Hum Reprod 33:464–473. 29304224 10.1093/humrep/dex377

[8] Bothun AM, Y Gao, Y Takai, O Ishihara, H Seki, B Karger, JL Tilly and DC Woods. (2018). Quantitative proteomic profiling of the human ovary from early to mid-gestation reveals protein expression dynamics of oogenesis and folliculogenesis. Stem Cells Dev 27:723–735. 29631484 10.1089/scd.2018.0002 PMC5985909

[9] Clarkson YL, M McLaughlin, M Waterfall, CE Dunlop, PA Skehell, RA Anderson and EE Telfer. (2018). Initial characterisation of adult human ovarian cell populations isolated by DDX4 expression and aldehyde dehydrogenase activity. Sci Rep 8:6953. 29725036 10.1038/s41598-018-25116-1 PMC5934371

[10] MacDonald JA, Y Takai, O Ishihara, H Seki, DC Woods and JL Tilly. (2019). Extracellular matrix signaling activates differentiation of adult ovary-derived oogonial stem cells in a species-specific manner. Fertil Steril 111:794–805. 30871765 10.1016/j.fertnstert.2018.12.015 PMC6462309

[11] Sequeira RC, S Sittadjody, T Criswell, A Atala, JD Jackson and JJ Yoo. (2021). Enhanced method to select human oogonial stem cells for fertility research. Cell Tissue Res 386:145–156. 34415395 10.1007/s00441-021-03464-1

[12] Ariyath A, MK Janu and B Paul-Prasanth. (2022). Differentiation potential of cultured extracellular DEAD-box helicase 4+ oogonial stem cells from adult human ovaries into somatic lineages. Cells Tissues Organs 211:577–588. 34412061 10.1159/000519087

[13] Wu M, Z Lu, Q Zhu, L Ma, L Xue, Y Li, S Zhou, W Yan, W Ye, et al. (2022). DDX04+ stem cells in the ovaries of postmenopausal women: existence and differentiation potential. Stem Cells 40:88–101. 35511860 10.1093/stmcls/sxab002

[14] Alberico H, Z Fleischmann, T Bobbitt, Y Takai, O Ishihara, H Seki, RA Anderson, EE Telfer, DC Woods and JL Tilly. (2022). Workflow optimization for identification of female germline or oogonial stem cells in human ovarian cortex using single-cell RNA sequence analysis. Stem Cells 40:523–536. 35263439 10.1093/stmcls/sxac015 PMC9199849

[15] Saber M, P Tavakol and F Esfandiari. (2022). Isolation of female germline stem cells from mouse and human ovaries by differential adhesion. Int J Cell Biol 2022:5224659. 36120418 10.1155/2022/5224659 PMC9473869

[16] Zou K, Z Yuan, Z Yang, H Luo, K Sun, L Zhou, J Xiang, L Shi, Q Yu, et al. (2009). Production of offspring from a germline stem cell line derived from neonatal ovaries. Nat Cell Biol 11:631–636. 19363485 10.1038/ncb1869

[17] Zhang Y, Z Yang, Y Yang, S Wang, L Shi, W Xie, K Sun, K Zou, L Wang, et al. (2011). Production of transgenic mice by random recombination of targeted genes in female germline stem cells. J Mol Cell Biol 3:132–141. 21149239 10.1093/jmcb/mjq043

[18] Zhou L, L Wang, JX Kang, W Xie, X Li, C Wu, B Xu and J Wu. (2014). Production of *fat-1* transgenic rats using a post-natal female germline stem cell line. Mol Hum Reprod 20:271–281. 24258451 10.1093/molehr/gat081

[19] Xiong J, Z Lu, M Wu, J Zhang, J Cheng, A Luo, W Shen, L Fang, S Zhou and S Wang. (2015). Intraovarian transplantation of female germline stem cells rescues ovarian function in chemotherapy-injured ovaries. PLoS One 10:e0139824. 26431320 10.1371/journal.pone.0139824 PMC4592213

[20] Zhang C and J Wu. (2016). Production of offspring from a germline stem cell line derived from prepubertal ovaries of germline reporter mice. Mol Hum Reprod 22:457–464. 27141102 10.1093/molehr/gaw030

[21] Wang N, C Satirapod, Y Ohguchi, ES Park, DC Woods and JL Tilly. (2017). Genetic studies in mice directly link oocytes produced during adulthood to ovarian function and natural fertility. Sci Rep 7:10011. 28855574 10.1038/s41598-017-10033-6 PMC5577229

[22] Wu C, B Xu, X Li, W Ma, P Zhang, X Chen and J Wu. (2017). Tracing and characterizing the development of transplanted female germline stem cells in vivo. Mol Ther 25:1408–1419. 28528817 10.1016/j.ymthe.2017.04.019 PMC5475279

[23] Li X, M Zheng, B Xu, D Li, Y Shen, Y Nie, L Ma and J Wu. (2021). Generation of offspring-producing 3D ovarian organoids derived from female germline stem cells and their application in toxicological detection. Biomaterials 279:121213. 34715637 10.1016/j.biomaterials.2021.121213

[24] Guo K, C-H Li, X-Y Wang, D-J He and P Zheng. (2016). Germ stem cells are active in postnatal mouse ovary under physiological conditions. Mol Hum Reprod 22:316–328. 26916381 10.1093/molehr/gaw015 PMC4847614

[25] Niikura Y, T Niikura and JL Tilly. (2009). Aged mouse ovaries possess rare premeiotic germ cells that can generate oocytes following transplantation into a young host environment. Aging (Albany, NY) 1:971–978. 20157580 10.18632/aging.100105 PMC2815754

[26] Stolzenbach V, DC Woods and JL Tilly. (2022). Non-neutral clonal selection and its potential role in mammalian germline stem cell dysfunction with advancing age. Front Cell Dev Biol 10:942652. 36081905 10.3389/fcell.2022.942652 PMC9445274

[27] Goodell MA, H Nguyen and N Shroyer. (2015). Somatic stem cell heterogeneity: diversity in the blood, skin and intestinal stem cell compartments. Nat Rev Mol Cell Biol 16:299–309. 25907613 10.1038/nrm3980 PMC5317203

[28] Dykstra B, D Kent, M Bowie, L McCaffrey, M Hamilton, K Lyons, SJ Lee, R Brinkman and C Eaves. (2007). Long-term propagation of distinct hematopoietic differentiation programs in vivo. Cell Stem Cell 1:218–229. 18371352 10.1016/j.stem.2007.05.015

[29] Dulken BW, DS Leeman, SC Boutet, K Hebestreit and A Brunet. (2017). Single-cell transcriptomic analysis defines heterogeneity and transcriptional dynamics in the adult neural stem cell lineage. Cell Rep 18:777–790. 28099854 10.1016/j.celrep.2016.12.060 PMC5269583

[30] Vicinanza C, I Aquila, M Scalise, F Cristiano, F Marino, E Cianflone, T Mancuso, P Marotta, W Sacco, FC Lewis and L Couch. (2017). Adult cardiac stem cells are multipotent and robustly myogenic: c-kit expression is necessary but not sufficient for their identification. Cell Death Differ 24:2101–2116. 28800128 10.1038/cdd.2017.130 PMC5686347

[31] Moreb JS. (2008). Aldehyde dehydrogenase as a marker for stem cells. Curr Stem Cell Res Ther 3:237–246. 19075754 10.2174/157488808786734006

[32] Zhao D, P McCaffery, KJ Ivins, RL Neve, P Hogan, WW Chin and UC Dräger. (1996). Molecular identification of a major retinoic-acid-synthesizing enzyme, a retinaldehyde-specific dehydrogenase. Eur J Biochem 240:15–22. 8797830 10.1111/j.1432-1033.1996.0015h.x

[33] Vassalli G. (2019). Aldehyde dehydrogenases: not just markers, but functional regulators of stem cells. Stem Cells Int 2019:3904645. 30733805 10.1155/2019/3904645 PMC6348814

[34] Anderson R, R Fassler, E Georges-Labouesse, RO Hynes, BL Bader, JA Kreidberg, K Schaible, J Heasman and C Wylie. (1999). Mouse primordial germ cells lacking β1 integrins enter the germline but fail to migrate normally to the gonads. Development 126:1655–1664. 10079228 10.1242/dev.126.8.1655

[35] Evans JP, RM Schultz and GS Kopf. (1995). Identification and localization of integrin subunits in oocytes and eggs of the mouse. Mol Reprod Dev 40:211–220. 7766414 10.1002/mrd.1080400210

[36] Burns KH, GE Owens, JM Fernandez, JH Nilson and MM Matzuk. (2002). Characterization of integrin expression in the mouse ovary. Biol Reprod 67:743–751. 12193380 10.1095/biolreprod.101.000729

[37] Li B, W Liu, M Zhuang, N Li, S Wu, S Pan and J Hua. (2016). Overexpression of CD61 promotes hUC-MSC differentiation into male germ-like cells. Cell Prolif 49:36–47. 26840189 10.1111/cpr.12236 PMC6496844

[38] Hayashi K, H Ohta, K Kurimoto, S Aramaki and M Saitou. (2011). Reconstitution of the mouse germ cell specification pathway in culture by pluripotent stem cells. Cell 146:519–532. 21820164 10.1016/j.cell.2011.06.052

[39] Navaroli D, JL Tilly and DC Woods. (2016). Isolation of mammalian oogonial stem cells by antibody-based fluorescence-activated cell sorting. Methods Mol Biol 1457:253–268. 27557587 10.1007/978-1-4939-3795-0_19 PMC8802829

[40] Zhang H, S Panula, S Petropoloulos, D Edsgärd, K Busayavalasa, L Liu, X Li, S Risal, Y Shen, et al. (2015). Adult human and mouse ovaries lack DDX4-expressing functional oogonial stem cells. Nat Med 21:1116–1118. 26444631 10.1038/nm.3775

[41] Wagner M, M Yoshihara, I Douagi, A Damdimopoulos, S Panula, S Petropoulos, H Lu, K Pettersson, K Palm, et al. (2020). Single-cell analysis of human ovarian cortex identifies distinct cell populations but no oogonial stem cells. Nat Commun 11:1147. 32123174 10.1038/s41467-020-14936-3 PMC7052271

[42] Yoshihara M, M Wagner, A Damdimopoulos, C Zhao, S Petropoulos, S Katayama, J Kere, F Lanner and P Damdimopoulou. (2022). The continued absence of functional germline stem cells in adult ovaries. Stem Cells 25:sxac070. 10.1093/stmcls/sxac070 PMC9982068 36153824

[43] Woods DC and JL Tilly. (2015). Reply to adult human and mouse ovaries lack DDX4-expressing functional oogonial stem cells. Nat Med 21:1118–1121. 26444631 10.1038/nm.3775

[44] Woods DC and JL Tilly. (2022). Revisiting claims of the continued absence of functional germline stem cells in adult ovaries. Stem Cells 6:sxac083. 10.1093/stmcls/sxac083 PMC9982064 36472569

[45] Fujiwara Y, T Komiya, H Kawabata, M Sato, H Fujimoto, M Furusawa and T Noce. (1994). Isolation of a DEAD-family protein gene that encodes a murine homolog of *Drosophila vasa* and its specific expression in germ cell lineage. Proc Natl Acad Sci U S A 91:12258–12262. 7991615 10.1073/pnas.91.25.12258 PMC45416

[46] Toyooka Y, N Tsunekawa, Y Takahashi, Y Matsui, M Satoh and T Noce. (2000). Expression and intracellular localization of mouse Vasa-homologue protein during germ cell development. Mech Dev 93:139–149. 10781947 10.1016/s0925-4773(00)00283-5

[47] Richards M, C-Y Fong and A Bongso. (2010). Comparative evaluation of different in vitro systems that stimulate germ cell differentiation in human embryonic stem cells. Fertil Steril 93:986–994. 19064262 10.1016/j.fertnstert.2008.10.030

[48] Yu DCW, FC Wu, CE Wu, LP Chow, HN Ho and HF Chen. (2020). Human pluripotent stem cell derived DDX4 and KRT-8 positive cells participate in ovarian follicle-like structure formation. iScience 24:102003. 33490911 10.1016/j.isci.2020.102003 PMC7811146

[49] Fox N, I Damjanov, A Martinez-Hernandez, BB Knowles and D Solter. (1981). Immunohistochemical localization of the early embryonic antigen (SSEA-1) in postimplantation mouse embryos and fetal and adult tissues. Dev Biol 83:391–398. 6113181 10.1016/0012-1606(81)90487-5

[50] Feng CW, J Bowles and P Koopman. (2014). Control of mammalian germ cell entry into meiosis. Mol Cell Endocrinol 382:488–497. 24076097 10.1016/j.mce.2013.09.026

[51] Wang N and JL Tilly. (2010). Epigenetic status determines germ cell meiotic commitment in embryonic and postnatal mammalian gonads. Cell Cycle 9:339–349. 20009537 10.4161/cc.9.2.10447 PMC5549632

[52] Satirapod C, N Wang, JA MacDonald, M Sun, DC Woods and JL Tilly. (2020). Estrogen regulation of female germline stem cell differentiation as a mechanism contributing to female reproductive aging. Aging (Albany, NY) 12:7313–7333. 32302290 10.18632/aging.103080 PMC7202493

[53] Jin C, X Wang, AD Hudgins, A Gamliel, M Pei, S Kim, D Contreras, J Hoeijmakers, J Campisi, et al. (2022). The regulatory landscapes of human ovarian aging. BioRxiv; DOI: 10.1101/2022.5.18.492547.

[54] Erler P, A Sweeney and JR Monaghan. (2017). Regulation of injury-induced ovarian regeneration by activation of oogonial stem cells. Stem Cells 35:236–247. 28028909 10.1002/stem.2504

[55] Imudia AN, N Wang, Y Tanaka, YAR White, DC Woods and JL Tilly. (2013). Comparative gene expression profiling of adult mouse ovary-derived oogonial stem cells supports a distinct cellular identity. Fertil Steril 100:1451–1458. 23876535 10.1016/j.fertnstert.2013.06.036 PMC4270279

[56] Park ES, DC Woods and JL Tilly. (2103). Bone morphogenetic protein 4 (BMP4) promotes mammalian oogonial stem cell differentiation via SMAD1/5/8 signaling. Fertil Steril 100:1468–1475. 10.1016/j.fertnstert.2013.07.1978 PMC4266321 23993924

[57] Moreb JS, D Ucar, S Han, JK Amory, AS Goldstein, B Ostmark and LJ Chang. (2012). The enzymatic activity of human aldehyde dehydrogenases 1A2 and 2 (ALDH1A2 and ALDH2) is detected by Aldefluor, inhibited by diethylaminobenzaldehyde and has significant effects on cell proliferation and drug resistance. Chem Biol Interact 195:52–60. 22079344 10.1016/j.cbi.2011.10.007 PMC3350780

[58] Morris RJ and CS Potten. (1994). Slowly cycling (label-retaining) epidermal cells behave like clonogenic stem cells in vitro. Cell Prolif 27:279–289. 10465012 10.1111/j.1365-2184.1994.tb01425.x

[59] Kenney NJ, GH Smith, E Lawrence, JC Barrett and DS Salomon. (2001). Identification of stem cell units in the terminal end bud and duct of the mouse mammary gland. J Biomed Biotechnol 1:133–143. 12488607 10.1155/S1110724301000304 PMC129060

[60] Braun KM, C Niemann, UB Jensen, JP Sundberg, V Silva-Vargas and FM Watt. (2003). Manipulation of stem cell proliferation and lineage commitment: visualisation of label-retaining cells in wholemounts of mouse epidermis. Development 130:5241–5255. 12954714 10.1242/dev.00703

[61] Tumbar T, G Guasch, V Greco, C Blanpain, WE Lowry, M Rendl and E Fuchs. (2003). Defining the epithelial stem cell niche in skin. Science 303:359–363. 14671312 10.1126/science.1092436 PMC2405920

[62] Oliver JA, O Maarouf, FH Cheema, TP Martens and Q Al-Awqati. (2004). The renal papilla is a niche for adult kidney stem cells. J Clin Invest 114:795–804. 15372103 10.1172/JCI20921 PMC516259

[63] Saitou M, S Barton and M Surani. (2002). A molecular programme for the specification of germ cell fate in mice. Nature 418:293–300. 12124616 10.1038/nature00927

[64] Ohinata Y, B Payer, D O'Carroll, K Ancelin, Y Ono, M Sano, SC Barton, T Obukhanych, M Nussenzweig, et al. (2005). Blimp1 is a critical determinant of the germ cell lineage in mice. Nature 436:207–213. 15937476 10.1038/nature03813

[65] Dolci S, L Levati, M Pellegrini, I Faraoni, G Graziani, A Di Carlo and R Geremia. (2002). Stem cell factor activates telomerase in mouse mitotic spermatogonia and in primordial germ cells. J Cell Sci 115:1643–1649. 11950883 10.1242/jcs.115.8.1643

[66] Durcova-Hills G, T Tokunaga, S Kurosaka, M Yamaguchi, S Takahashi and H Imai. (1999). Immunomagnetic isolation of primordial germ cells and the establishment of embryonic germ cell lines in the mouse. Cloning 1:217–224. 16218822 10.1089/15204559950019852

[67] Hou C, X Zhao, GG Tian and J Wu. (2022). *Stella* regulates the development of female germline stem cells by modulating chromatin structure and DNA methylation. Int J Biol Sci 18:3006–3018. 35541912 10.7150/ijbs.69240 PMC9066111

[68] Heyting C. (1996). Synaptonemal complexes: structure and function. Curr Opin Cell Biol 8:389–396. 8743892 10.1016/s0955-0674(96)80015-9

[69] Page SL and RS Hawley. (2004). The genetics and molecular biology of the synaptonemal complex. Ann Rev Cell Dev Biol 20:525–558. 15473851 10.1146/annurev.cellbio.19.111301.155141

[70] Prieto I, J Suja, N Pezzi, L Kremmer, C Martínez-A, JS Rufus and JL Barbero. (2001). Mammalian STAG3 is a cohesin specific to sister chromatid arms in meiosis I. Nat Cell Biol 3:761–766. 11483963 10.1038/35087082

[71] Hopkins J, G Hwang, J Jacob, J,N Sapp, R Bedigian, K Oka, P Overbeek, S Murray and PW Jordan. (2014). Meiosis-specific cohesin component, Stag3 is essential for maintaining centromere chromatid cohesion, and required for DNA repair and synapsis between homologous chromosomes. PLoS Genet 10:e1004413. 24992337 10.1371/journal.pgen.1004413 PMC4081007

[72] Winters T, F McNicoll and R Jessberger. (2014). Meiotic cohesin STAG3 is required for chromosome axis formation and sister chromatid cohesion. EMBO J 33:1256–1270. 24797474 10.1002/embj.201387330 PMC4198028

[73] Ishiguro KI. (2019). The cohesin complex in mammalian meiosis. Genes Cells 24:6–30. 30479058 10.1111/gtc.12652 PMC7379579

[74] Parisi S, MJ McKay, M Molnar, MA Thompson, PJ van der Spek, E van Drunen-Schoenmaker, R Kanaar, E Lehmann, JH Hoeijmakers and J Kohli. (1999). Rec8p, a meiotic recombination and sister chromatid cohesion phosphoprotein of the Rad21p family conserved from fission yeast to humans. Mol Cell Biol 19:3515–3528. 10207075 10.1128/mcb.19.5.3515 PMC84144

[75] Hayashi K, S Ogushi, K Kurimoto, S Shimamoto, H Ohta and M Saitou. (2012). Offspring from oocytes derived from in vitro primordial germ cell-like cells in mice. Science 338:971–975. 23042295 10.1126/science.1226889

[76] Hikabe O, N Hamazaki, G Nagamatsu, Y Obata, Y Hirao, N Hamada, S Shimamoto, T Imamura, K Nakashima, M Saitou and K Hayashi. (2016). Reconstitution in vitro of the entire cycle of the mouse female germ line. Nature 539:299–303. 27750280 10.1038/nature20104

[77] Hayashi K, O Hikabe, Y Obata and Y Hirao. (2017). Reconstitution of mouse oogenesis in a dish from pluripotent stem cells. Nat Protoc 12:1733–1744. 28796232 10.1038/nprot.2017.070

[78] Selesniemi K, HJ Lee and JL Tilly. (2008). Moderate caloric restriction initiated in rodents during adulthood sustains function of the female reproductive axis into advanced chronological age. Aging Cell 7:622–629. 18549458 10.1111/j.1474-9726.2008.00409.x PMC2990913

[79] Selesniemi K, HJ Lee, T Niikura and JL Tilly. (2008). Young adult donor bone marrow infusions into female mice postpone age-related reproductive failure and improve offspring survival. Aging (Albany NY) 1:49–57. 20157587 10.18632/aging.100002 PMC2815764

[80] Selesniemi K, HJ Lee, A Muhlhauser and JL Tilly. (2011). Prevention of maternal aging-associated oocyte aneuploidy and meiotic spindle defects in mice by dietary and genetic strategies. Proc Natl Acad Sci U S A 108:12319–12324. 21730149 10.1073/pnas.1018793108 PMC3145697

[81] Le Quesne Stabej P, HJ Williams, C James, M Tekman, HC Stanescu, R Kleta, L Ocaka, F Lescai, HL Storr, et al. (2016). *STAG3* truncating variant as the cause of primary ovarian insufficiency. Eur J Hum Genet 24:135–138. 26059840 10.1038/ejhg.2015.107 PMC4795223

[82] Jaillard S, K McElreavy, G Robevska, L Akloul, F Ghieh, R Sreenivasan, M Beaumont, A Bashamboo, J Bignon-Topalovic, et al. (2020). *STAG3* homozygous missense variant causes primary ovarian insufficiency and male non-obstructive azoospermia. Mol Hum Reprod 26:665–677. 32634216 10.1093/molehr/gaaa050

[83] Mellone S, M Zavattaro, D Vurchio, S Ronzani, M Caputo, I Leone, F Prodam and M Giordano. (2021). A long contiguous stretch of homozygosity disclosed a novel *STAG3* biallelic pathogenic variant causing primary ovarian insufficiency: a case report and review of the literature. Genes (Basel) 12:1709. 34828315 10.3390/genes12111709 PMC8622734

[84] Schleiffer A, S Kaitna, S Maurer-Stroh, M Glotzer, K Nasmyth and F Eisenhaber. (2003). Kleisins: a superfamily of bacterial and eukaryotic SMC protein partners. Mol Cell 11:571–575. 12667442 10.1016/s1097-2765(03)00108-4

[85] Garcia-Cruz R, MA Brieño, I Roig, M Grossmann, E Velilla, A Pujol, L Cabero, A Pessarrodona, JL Barbero and M Garcia Caldés. (2010). Dynamics of cohesin proteins REC8, STAG3, SMC1β and SMC3 are consistent with a role in sister chromatid cohesion during meiosis in human oocytes. Hum Reprod 25:2316–2327. 20634189 10.1093/humrep/deq180

[86] Bouilly J, I Beau, S Barraud, V Bernard, K Azibi, J Fagart, A Fèvre, AL Todeschini, RA Veitia, et al. (2016). Identification of multiple gene mutations accounts for a new genetic architecture of primary ovarian insufficiency. J Clin Endocrinol Metab 101:4541–4550. 27603904 10.1210/jc.2016-2152

[87] Beverley R, ML Snook and MA Brieño-Enríquez. (2021). Meiotic cohesin and variants associated with human reproductive aging and disease. Front Cell Dev Biol 9:710033. 34409039 10.3389/fcell.2021.710033 PMC8365356

[88] Tilly JL and DC Woods. (2021). Reproductive medicine at the crossroads of stem cell biology and big data. Fertil Steril 116:686–687. 34294453 10.1016/j.fertnstert.2021.06.042

[89] Richardson GM, J Lannigan and IG Macara. (2015). Does FACS perturb gene expression? Cytometry A 87:166–175. 25598345 10.1002/cyto.a.22608

