# Supplementary material for: Characterization of Oogonial Stem Cells in Adult Mouse Ovaries with Age and Comparison to In Silico Data on Human Ovarian Aging
Source: Stem Cells Dev. 2023 Mar 3;32(5-6):99–114. doi: 10.1089/scd.2022.0284 (PMC9986025; doi:10.1089/scd.2022.0284)
Supplement: Supplemental data [file Supp_TableS1.docx]

**SUPPLEMENTAL TABLE 1.** Sequences of primers used for conventional PCR.

| **Gene name** | **Primer Sequence (5'→3')** | **Amplicon (bp)** |
| --- | --- | --- |
| *Prdm1* | Forward: GTGCCCTACCAAGAAGGATTT | 784 |
|  | Reverse: AAGAGGAAGAGGAAGAGGAGAG | |
| *Dppa3* | Forward: CCCAATGAAGGACCCTGAAA | 571 |
|  | Reverse: GGATCGTTGTGCATCCTATCT |  |
| *Ifitm3* | Forward: CCATCCTTTGCCCTTCAGT | 315 |
|  | Reverse: GTCACATCACCCACCATCTT |  |
| *Tert* | Forward: AGATTGGTGACAGGTGCTATAC | 425 |
|  | Reverse: GAAGGTGAGGCTCGTCTTAAT |  |
| *β-actin* | Forward: GACAGGATGCAGAAGGAGATTAC | 791 |
|  | Reverse: GGGAGACCAAAGCCTTCATAC |  |
