# Supplementary material for: Characterization of Oogonial Stem Cells in Adult Mouse Ovaries with Age and Comparison to In Silico Data on Human Ovarian Aging
Source: Stem Cells Dev. 2023 Mar 3;32(5-6):99–114. doi: 10.1089/scd.2022.0284 (PMC9986025; doi:10.1089/scd.2022.0284)
Supplement: Supplemental data [file Supp_TableS2.docx]

**SUPPLEMENTAL TABLE 2.** Sequences of primers used for quantitative PCR.

| **Gene name** | **Primer Sequence (5'→3')** | | **Amplicon (bp)** |
| --- | --- | --- | --- |
| *Prdm1* | Forward: CGGAAAGCAACCCAAAGCAATAC | | 483 |
|  | Reverse: CCTCGGAACCATAGGAAACATTC | |  |
| *Dppa3* | Forward: CCCAATGAAGGACCCTGAAAC | | 354 |
|  | Reverse: AATGGCTCACTGTCCCGTTCA | |  |
| *Ifitm3* | Forward: GTTATCACCATTGTTAGTGTCATC | | 151 |
|  | Reverse: AATGAGTGTTACACCTGCGTG | |  |
| *Tert* | Forward: TGCCAATATGATCAGGCACTCG | | 305 |
|  | Reverse: ACTGCGTATAGCACCTGTCACC | |  |
| *β2-microglobulin* | Forward: TTCTGGTGCTTGTCTCACTGA | | 104 |
|  | Reverse: CAGTATGTTCGGCTTCCCATTC |  | |
